# NICA: A Novel Toolbox for Near-Infrared Spectroscopy Calculations and Analyses

**DOI:** 10.3389/fninf.2020.00026

**Published:** 2020-05-25

**Authors:** Philipp Raggam, Günther Bauernfeind, Selina C. Wriessnegger

**Affiliations:** ^1^Institute of Neural Engineering, Graz University of Technology, Graz, Austria; ^2^Independent Researcher, Hanover, Germany; ^3^BioTechMed-Graz, Graz, Austria

**Keywords:** functional near-infrared spectroscopy, hemodynamic responses, signal processing, physiological artifact correction, MATLAB toolbox, graphical user interface, NIRScout

## Abstract

Functional near-infrared spectroscopy (fNIRS) measures the functional activity of the cerebral cortex. The concentration changes of oxygenated (oxy-Hb) and deoxygenated hemoglobin (deoxy-Hb) can be detected and associated with activation of the cortex in the investigated area (neurovascular coupling). Recorded signals of hemodynamic responses may contain influences from physiological signals (systemic influences, physiological artifacts) which do not originate from the cerebral cortex activity. The physiological artifacts contain the blood pressure (BP), respiratory patterns, and the pulsation of the heart. In order to perform a comprehensive analysis of recorded fNIRS data, a proper correction of these physiological artifacts is necessary. This article introduces NICA – a novel toolbox for near-infrared spectroscopy calculations and analyses based on MATLAB. With NICA it is possible to process and visualize fNIRS data, including different signal processing methods for physiological artifact correction. The artifact correction methods used in this toolbox are common average reference (CAR), independent component analysis (ICA), and transfer function (TF) models. A practical example provides results from a study, where NICA was used for analyzing the measurement data, in order to demonstrate the signal processing steps and the physiological artifact correction. The toolbox was developed for fNIRS data recorded with the NIRScout 1624 measurement device and the corresponding recording software NIRStar.

## Introduction

Functional near-infrared spectroscopy (fNIRS) is a non-invasive optical method for detecting functional hemodynamic activity of the cerebral cortex. This method measures the regional changes of the oxygen concentration in the blood, more precisely the concentration changes of oxygenated (oxy-Hb) and deoxygenated hemoglobin (deoxy-Hb; Jöbsis, 1977; Wolf et al., 2007; Scholkmann et al., 2014). These concentration changes are coupled with neuronal activity (i.e., neurovascular coupling) and can therefore be associated with activation of the cortex in the investigated area during certain cognitive and motor tasks (Villringer et al., 1993; Leithner and Royl, 2014).

In order to measure the concentration changes of oxy-Hb and deoxy-Hb, near-infrared light is sent from and detected by optical sensors (sources and detectors), so called optodes. The near-infrared light can penetrate the cranial bone, and the photons are absorbed and scattered by different interactions with the cerebral tissue. The scattered photons follow a random path through the tissue, where parts of these photons get absorbed (e.g., by chromophores such as oxy-Hb and deoxy-Hb). Some of the photons are back-scattered and can be measured by detectors which are placed at a distance of a few centimeters (∼3 cm) around the source (Okada et al., 1997; Boas et al., 2004; Ferrari and Quaresima, 2012). Using the (modified) Beer–Lambert law, it is possible to calculate the concentration changes in oxy-Hb and deoxy-Hb according to the attenuation of the intensity of the near-infrared light between the source and the detector (Coyle et al., 2007). With these concentration changes, conclusions about metabolic changes in the investigated area can be drawn, which are associated with brain activity (Malonek and Grinvald, 1996; Wolf et al., 2002).

Aside from brain activity, the recorded signals of the hemodynamic responses may contain influences from physiological signals which do not originate from the cerebral cortex (systemic influences) (Coyle et al., 2004; Takahashi et al., 2011; Tachtsidis and Scholkmann, 2016). These signals include the pulsation of the heart, the respiratory patterns and low frequency oscillations from the blood pressure (BP; De Boer et al., 1986; Koepchen, 1991). The frequency of the pulse waves (heart rate, HR) typically lies between 1 and 2 Hz, the respiratory frequency (RF) is in a range from 0.2 to 0.4 Hz (Bauernfeind et al., 2014). The third order BP waves, also known as Mayer–Traube–Hering (MTH) waves, occur between 0.07 and 0.13 Hz (Pfurtscheller et al., 2010b). Since the time constant of hemodynamic activation responses is about 5–10 s (Elwell et al., 1999), the systemic influences, especially the MTH waves and the RF, may influence and superimpose the recorded cortical activation (Obrig et al., 2000; Bauernfeind et al., 2008).

A number of methods have been proposed to reduce the interfering physiological signals, such as different types of low-pass filters (Bauernfeind et al., 2008; Power et al., 2011), moving average filters (Soraghan et al., 2008), as well as pulse regression (Gratton and Corballis, 1995) and spatial filtering (Pfurtscheller et al., 2010a). Although these methods can be very useful to remove physiological noise, they can also cause a reduction of the hemodynamic responses itself (Bauernfeind et al., 2014). Therefore, it is required that the systemic influences in the frequency range of the fNIRS signal are detected and removed. However, most studies show that these influences and their removal are neglected (Leff et al., 2011). Bauernfeind et al. (2014) proposed three different signal processing methods that can help to reduce the systemic influences of the recorded fNIRS-signal: a common average reference (CAR) method, independent component analysis (ICA) and the use of transfer function (TF) models. Therefore, it is necessary to simultaneously record the physiological signals (BP, RF, and HR).

To perform a thorough analysis of fNIRS data, it is necessary to pre-process the data, remove physiological and technical artifacts, calculate the concentration changes of oxy-Hb and deoxy-Hb and generate output files and figures to visualize the recorded signals. There are freely available toolboxes or compact software packages to analyze fNIRS data, such as Homer2 (Huppert et al., 2009), NIRS-SPM (Watanabe et al., 2018), or the NIRS Brain AnalyzIR Toolbox (Santosa et al., 2018). However, these toolboxes and software packages do not offer artifact correction methods for fNIRS data using simultaneously recorded physiological signals. For this purpose, the MATLAB toolbox NICA was developed – a graphical user interface (GUI), which offers a user-friendly handling of processing and visualizing fNIRS data, including all the necessary signal processing steps and the correction of physiological and technical artifacts.

With NICA it is possible to load and pre-process the fNIRS data, apply correction methods for physiological artifacts (systemic influences), exclude noisy channels and/or trials, and set different options for visualizing the results of the analysis. This article focuses on the implemented signal processing and physiological artifact correction, the implementation and handling of the GUI, as well as on the requirements for the measurement system and data. Furthermore, an example analysis will be provided, showing the importance of proper artifact correction.

## Materials and Methods

### FNIRS Measurement Device and File Requirements

NICA was designed and optimized to analyze measurement data recorded with the NIRScout 1624 device and its corresponding recording software NIRStar (NIRx Medizintechnik GmbH, Berlin, Germany). This measurement system uses a multichannel continuous wave (CW) technique with a sampling rate of 3.91 Hz. It offers 16 sources, where near-infrared light is emitted at a wavelength of 760 and 850 nm, and 24 detectors. In order to perform an analysis, two file formats are required for the software: a header-file (HDR-file) and an extensible data format file (XDF-file).

The HDR-file provides basic information about the measurement setup (filename, date, number of sources and detectors, etc.) and is generated with the NIRStar recording software (NIRx Medizintechnik GmbH, Berlin, Germany), which is used to measure the fNIRS signals. This software is generally used and delivered with the NIRScout device.

The XDF-file includes all the recorded signals of a measurement: fNIRS data, electrocardiogram (ECG), HR, BP, RF as well as the paradigm markers. The signals are streamed and synchronized in time via lab streaming layer (LSL) applications (Kothe, 2014) and recorded with the LabRecorder (default recording program for the LSL). The fNIRS signals are recorded with two different wavelengths, 760 and 850 nm, using the CW approach. ECG and RF are measured with surface electrodes and a stretching sensor in a chest strap (Respiratory Effort Sensor, Pro-Tech Services, United States), respectively, at a sampling rate of 256 Hz, and connected via a biosignal amplifier (g.USBamp, Guger Technologies, Austria) to the LSL application. HR and BP are recorded with a finger and an upper arm cuff with the CNAP Monitor 500 (CNSystems Medizintechnik AG, Austria). The CNAP device measures the blood pressure continuously, noninvasively and beat-to-beat on the finger artery with a sampling rate of 200 Hz. The upper arm measurement is used to calibrate the finger cuff to deliver absolute blood pressure values. The blood pressure values are displayed at the integrated monitor and sent via custom-made sync-box to the computer. The paradigm markers are sent during the paradigm presentation and are essential for the timing of the measurement (to distinguish between trials and different conditions). The physiological signals (ECG, HR, BP, and RF) are required for the artifact correction of the systemic influences. The ECG and the BP can both be used to correct the MTH-waves. If the CNAP Monitor is used, there is no need to measure the ECG as well. Even though an analysis can be performed without physiological signals, it is not recommended, since a thorough correction of the physiological artifacts is not possible.

### Signal Processing and Artifact Correction

The signal processing steps in NICA from the raw signals to the clean oxy-Hb and deoxy-Hb signals, as well as the physiological artifact removal methods, were introduced by Bauernfeind et al. (2014). This article focuses on the implementation and utilization of NICA; therefore, this section only roughly describes the usage and the parameters of the signal processing and artifact correction methods.

#### Removing 50 Hz Power Line Interference

In the first signal processing step, a filter is applied on the ECG and the respiratory signal to remove the 50 Hz power line interference. The used filter is an infinite impulse response (IIR) notch filter (band-stop) with a cut-off frequency of 50 Hz and a bandwidth of 1.43 Hz. The removing of the power line interference of the other signals (fNIRS, BP, and HR) is included in their measurement device.

#### Calculating Concentration Changes of oxy-Hb and deoxy-Hb

The NIRScout measurement device uses the CW approach, which detects the attenuation of light as their only measurement parameter. The attenuation (*A*) can be calculated with Eq 1 (Delpy and Cope, 1997):

(1) $$A=\log_{10}\left(\frac{I_{0}}{I}\right)=\left(\alpha_{H\,b}\cdot c_{H\,b}+\alpha_{H\,b\,O_{2}}\cdot c_{H\,b\,O_{2}}\right)\cdot x\cdot l+K$$

*A* is the attenuation of light (extinction), *I*_0_ the intensity emitted from the source, *I* the intensity measured from the detector, α*_*HbO*_*_2_ and α*_*Hb*_* are the molar coefficients of extinction of oxy-Hb and deoxy-Hb, *c*_*HbO2*_ and *c*_*Hb*_ the corresponding concentrations of the absorber (oxy-Hb and deoxy-Hb), *x* is the differential path length (DPF), *l* the geometric distance between source and detector, and *K* describes the tissue loss due to scattering. The DPF cannot be determined with the CW approach and has to be based on measured values. Hence, it is not possible to calculate absolute values of the concentration of oxy-Hb and deoxy-Hb, but rather concentration changes (in mM × mm) (Duncan et al., 1995).

The tissue loss can be seen as constant in a short time interval and therefore be neglected after differentiation:

(2) $$\Delta \,A=\log_{10}\left(\frac{I\,\left(t_{1}\right)}{I\,\left(t_{2}\right)}\right)=\left(\alpha_{H\,b}\cdot \Delta \,c_{H\,b}+\alpha_{H\,b\,O_{2}}\cdot \Delta \,c_{H\,b\,O_{2}}\right)\cdot x\cdot l$$

Rearranging Eq 2 leads to the following expression:

(3) $$\frac{\Delta \,A}{x\cdot l}=\alpha_{H\,b}\cdot \Delta \,c_{H\,b}+\alpha_{H\,b\,O_{2}}\cdot \Delta \,c_{H\,b\,O_{2}}$$

The calculation of the concentration changes of oxy-Hb and deoxy-Hb requires the two wavelengths with which the fNIRS signal was recorded (760 and 850 nm). Eq 3 can be used for both wavelengths and combined by using matrix notations:

(4) $$\left[\begin{array}{c} \frac{\Delta \,A_{760\,n\,m}}{x_{760\,n\,m}\cdot l}\\ \frac{\Delta \,A_{850\,n\,m}}{x_{850\,n\,m}\cdot l} \end{array}\right]=\left[\begin{array}{cc} \alpha_{760\,n\,m,H\,b}\; & \alpha_{760\,n\,m,H\,b\,O_{2}}\\ \alpha_{850\,n\,m,H\,b}\; & \alpha_{850\,n\,m,H\,b\,O_{2}} \end{array}\right]\cdot \left[\begin{array}{c} \Delta \,c_{H\,b}\\ \Delta \,c_{H\,b\,O_{2}} \end{array}\right]$$

After rearranging Eq 4, the concentration changes of oxy-Hb Δ*c*_*HbO*__2_ and deoxy-Hb Δ*c*_*Hb*_ can be calculated with Eq 5:

(5) $$\left[\begin{array}{c} \Delta \,c_{H\,b}\\ \Delta \,c_{H\,b\,O_{2}} \end{array}\right]={\left[\begin{array}{cc} \alpha_{760\,n\,m,H\,b}\; & \alpha_{760\,n\,m,H\,b\,O_{2}}\\ \alpha_{850\,n\,m,H\,b}\; & \alpha_{850\,n\,m,H\,b\,O_{2}} \end{array}\right]}^{-1}\cdot \left[\begin{array}{c} \frac{\Delta \,A_{760\,n\,m}}{x_{760\,n\,m}\cdot l}\\ \frac{\Delta \,A_{850\,n\,m}}{x_{850\,n\,m}\cdot l} \end{array}\right]$$

#### Removing Baseline Drifts From oxy-Hb and deoxy-Hb Signals

A 6th order Butterworth high-pass filter is applied to remove the baseline drifts from the oxy-Hb and deoxy-Hb signals. The cut-off frequency is 0.005 Hz, with a passband ripple of 1 dB and a stopband attenuation of 30 dB. The baseline drifts can be described as low frequency noise that shifts the baseline of the signal, which makes it difficult to detect characteristic features (Kaur and Singh, 2011).

#### Physiological Artifact Correction

As in Bauernfeind et al. (2014), three different signal processing methods are used by the toolbox that can help to reduce the physiological artifacts of the recorded fNIRS-signal: CAR, ICA, and TF models.

##### Common average reference

The CAR method is a simple technique to reduce noise (of technical or physiological origin) in the recorded signal, which has already been used for the analysis of EEG data (Zhang et al., 2007; Matthews et al., 2008; Ludwig et al., 2009). For analyzing fNIRS data, the CAR method was first introduced by Pfurtscheller et al. (2010a). This method uses the fact that systemic influences interfere with the signal in all channels, and can therefore be reduced by using the mean of all channels:

(6) $$N_{i}\,\left[n\right]=X_{i}\,\left[n\right]-\frac{1}{M}\,\sum_{j=1}^{M}X_{j}\,\left[n\right]$$

The new signal *N*_*i*_[*n*], as described in Eq 6, is calculated by subtracting the mean of all channels *X_*j*_, j = * 1 … *M* from each single channel *X*_*i*_, *i* = 1 … *M* for each time point *n*.

##### Independent component analysis

Independent component analysis is based on the mixing of a linear transformation Matrix *A* and the independent sources *S*, which results in the recorded and artifact influenced fNIRS signals *X* (Eq 7):

(7) X=A⁢S

The estimates (independent components, ICs) of the sources (oxy-Hb and deoxy-Hb signals with physiological artifacts) can be retained by using the inverse of the mixing matrix *A*^–1^. Assuming that the physiological artifacts are statistically independent from the oxy-Hb and deoxy-Hb signals, they can be divided into separate ICs and removed from the fNIRS signal. For the decomposition of the fNIRS signal into ICs, the Second Order Blind Identification (SOBI) implementation of the ICA algorithm is used (Belouchrani et al., 1997).

##### Transfer function models

The use of TF models was introduced by Florian and Pfurtscheller (1997) in order to remove respiratory arrhythmia at movement induced changes of the HR. In NICA, the TF models are applied to remove the perturbations of the physiological artifacts from the oxy-Hb and deoxy-Hb signals. The models can be described with Eq 8:

(8) $$X\,\left[n\right]=\sum_{u=0}^{m}g_{u}\,Y\,\left[n-u\right]+N\,\left[n\right]$$

*X*[*n*] describes the signal at each time point *n*, *Y*[*n*–*u*] the perturbation and *N*[*n*] the signal without influences. The parameters *g*_*u*_ can be estimated by minimizing the mean squared error *E*(*N*^2^[*n*]) (Florian and Pfurtscheller, 1997). After rearranging Eq 8, the signal without influences *N*[*n*] can be calculated with Eq 9:

(9) $$N\,\left[n\right]=X\,\left[n\right]-\sum_{u=0}^{m}g_{u}\,Y\,\left[n-u\right]$$

Either one of these artifact correction methods can be chosen for the analysis (the choice may also depend on the measurement data and if physiological signals are available). However, according to Bauernfeind et al. (2014) the TF models have proven to be the most effective method.

#### Applying a Low-Pass Filter

In order to remove further artifacts (of technical or physiological origin), there is also the option to apply a low-pass filter (if the artifact lies in a frequency range above the frequency of interest). The used filter is a Butterworth filter of 8th order with a passband ripple of 3 dB and a stopband attenuation of 30 dB. This filter can be applied if there are any distortions visible in the frequency spectrum (e.g., originating from technical artifacts). The cut-off frequency is freely selectable, depending on the frequency range of the distortion. If physiological artifact correction is applied, the filter should not interfere with the physiological signals, which means. the cut-off frequency should not be in the frequency range of the physiological signals.

#### Grand Average and Region-of-Interest Analysis

With NICA it is possible to run a grand average analysis on the group level. Therefore, the oxy-Hb and deoxy-Hb signals of individual subjects are averaged over all subjects at each channel. Eq 10 shows the calculations for the oxy-Hb signals:

(10) $$\Delta \,c_{H\,b}\,A\,V\,G=\frac{1}{N}\,\sum_{i=1}^{N}\Delta \,c_{H\,b}\,i$$

The matrix with the average concentration changes of oxy-Hb Δ*c_*Hb*_AVG* is calculated by summing up the individual concentration changes of oxy-Hb Δ*c_*Hb*_i* and then dividing them by the number of subjects *N*. The matrix notation is used, because the concentration change signals for all channels are calculated. The average concentration changes of deoxy-Hb are calculated in the same way.

At a grand average analysis, the oxy-Hb and deoxy-Hb signals from individual channels can be combined to a region-of-interest (ROI). The calculation of an ROI of the concentration changes of oxy-Hb is shown at Eq 11:

(11) $$\Delta \,c_{H\,b}\,R\,O\,I=\frac{1}{C}\,\sum_{c=1}^{C}\Delta \,c_{H\,b}\,c$$

The ROI of the concentration changes of oxy-Hb Δ*c_*Hb*_ROI*, as described at Eq 11, is calculated by summing up the concentration changes of oxy-Hb from individual channels Δ*c_*Hb*_c* and then dividing them by the number of channels *C.* The ROI of the concentration changes of deoxy-Hb is calculated in the same way.

## Implementation

### Overview and Requirements

NICA is available as a free and open MATLAB toolbox^1^. The toolbox includes the MATLAB files to install and start the GUI, the files to perform the analysis steps, as well as a directory for user settings and a user manual. The toolbox was developed and tested with the MATLAB releases 2015b, 2017a and 2019b. There are also additional software packages included, which are necessary for certain data handling and signal processing steps: BioSig v1.5 (Schlögl and Brunner, 2008) and EEGLAB v2008beta (Delorme and Makeig, 2004).

As mentioned in section “FNIRS Measurement Device and File Requirements,” an HDR-file and an XDF-file are required for NICA to perform an analysis. The XDF-file contains all the recorded signals of a measurement: fNIRS, ECG, HR, BP, RF as well as the paradigm markers. The paradigm markers must be set before the measurements and sent to the LabRecorder via LSL-stream. Without the markers it is not possible to average the fNIRS signals over trials (epochs) or distinguish between different conditions.

The toolbox can be installed by running the included installation file. After installation, the directory of the toolbox is added to the MATLAB paths, and NICA can simply be started by typing *nica* in the MATLAB command window.

### Surface of the GUI

**FIGURE 1:**
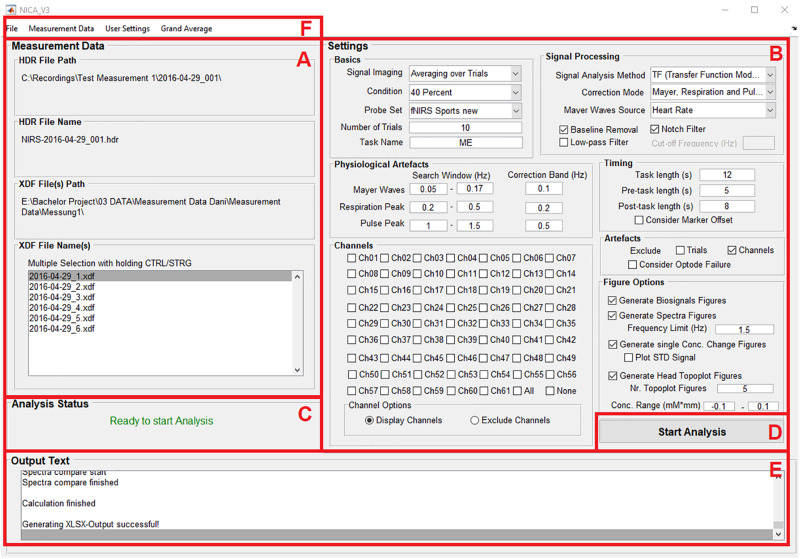
The surface of the GUI is divided into different panels: Information about the measurement data **(A)**; settings for the pre-processing of the measurement data, correction methods for physiological artifacts, excluding noisy channels or trials, and options for visualizing the results of the analysis **(B)**; information about the current analysis status **(C)**; a push button to start the analysis **(D)**; information about the individual analysis and signal processing steps **(E)**; and menu bar items allow the user to load the measurement data and save the analysis settings **(F)**.

As seen in Figure 1, the surface of the toolbox is divided into different panels: Information about the measurement data (A); settings for the pre-processing of the measurement data, correction methods for physiological artifacts, excluding noisy channels or trials, and options for visualizing the results of the analysis (B); information about the current analysis status (C); a push button to start the analysis (D); information about the individual analysis and signal processing steps (E); and menu bar items allow the user to load the measurement data and save the analysis settings (F).

The functions of the panels and menu items are described at Table 1. Detailed information about the handling of the GUI will be provided in section “Performing an Analysis” based on an example analysis.

**TABLE 1:** Description of the functions of the panels and menu items of NICA.

**Panel or menu item**	**Description**
Menu: File	Selecting and opening the current analysis path, closing all open figures or the GUI itself
Menu: Measurement Data	Loading the HDR- and XDF-files, displaying the file names and file paths at the corresponding panel
Menu: User Settings	Saving the settings in individual files, which can be loaded again for later use
Menu: Grand Average	Starting or stopping a grand average analysis (for all subjects), defining the regions of interest (combined channels)
Settings: Basics	Choosing the signal display method (continuous or averaged over trials), defining the conditions and the probe set and setting the number of trials and the task name
Settings: Signal Processing	Selecting the artifact correction method (CAR, ICA or TF models), the MTH-waves sources and additional signal processing steps (baseline removal, notch filter, low-pass filter)
Settings: Physiological Artifacts	Defining the frequency search window for the physiological artifacts and the correction band
Settings: Timing	Setting the timing for the visualization of the concentration change figures
Settings: Artifacts	Excluding artifact afflicted trials or channels, and considering an optode failure (interpolating with surrounding ones)
Settings: Channels	Displaying results for single channels individually or excluding single channels (if the exclude channels option is selected)
Settings: Figure Options	Selecting which figures are generated during the analysis (biosignals, frequency spectra, concentration change, topoplot) and setting the frequency limit and the concentration range
Analysis Status	Providing the current status of the analysis and information if an error occurs
Output Text	Showing detailed information about the processing steps in text format

### Performing an Analysis

In this section, the steps for performing an analysis of fNIRS data with NICA will be exemplified. The dataset for this example was recorded with the NIRScout 1624 measurement device and its corresponding recording software NIRStar. The experiment was divided into six runs; hence six HDR-files and six XDF-files were generated. With NICA, a single-run analysis, an analysis with several run-files combined, as well as a grand average analysis on the group level can be performed.

The participants had to perform either a motor execution (ME) task by applying force on a pressure sensor with the right hand, or a motor imagery (MI) task by imagining applying force on the pressure sensor. The force for both ME and MI tasks was instructed to be 20% (condition 1) or 40% (condition 2) of the participant’s maximum grip strength and had to be applied for 12 s. One trial consisted of a 5 s reference period (pre-task, looking at a reference cross), 12 s task time and 8 s resting period (looking at a black screen). In this example, a dataset with an ME task and 40% grip strength will be presented.

#### Selecting Analysis Path and Loading Measurement Data

Before an analysis can be started, the directory where the output-files will be saved has to be selected. After selecting the menu item *Select Analysis Path* at the menu bar entry *File* (Figure 1F), a dialog box opens where the preferred file path can be chosen. It is recommended, to use the directory of the raw XDF-files.

The raw XDF-files can be loaded under the menu bar entry *Measurement Data* (Figure 1F) with clicking on *Load* and then choosing *HDR File* and *XDF File(s)*. It is possible to load all XDF-files from a recording session (e.g., when the experiment is divided into different runs) and to perform the analysis for all files at once. When the measurement files are loaded, the file names and file paths are displayed on the left side of the GUI (Figure 1A).

#### Defining Conditions

Another important step is to define the different conditions (tasks) of your measurement data. The condition names and markers can be set under the menu bar entry *Measurement Data* by clicking on the option *Define Conditions.* There, the number of conditions must be entered and a name and a marker value for each condition must be defined (in this case 20% for condition 1 and 40% for condition 2). In order to average the measurement data over all trials correctly, the condition marker must fit the paradigm marker (i.e., same value), which are stored in the XDF-file.

#### Adjusting Analysis Settings

The analysis settings can be adjusted on the right side of the GUI. The settings are divided into different panels, according to their functionality (Figure 1B). Under the menu bar entry *User Settings* (Figure 1F) it is also possible to load a file with predefined analysis settings.

##### Basics

The basic settings include the signal imaging method (to display the oxy-Hb and deoxy-Hb signals averaged over trials or continuously), selecting one of the predefined conditions, setting the correct number of trials (of the selected condition) and defining a task name. The selected condition and the task name will be included in the name of the generated output files.

**FIGURE 2:**
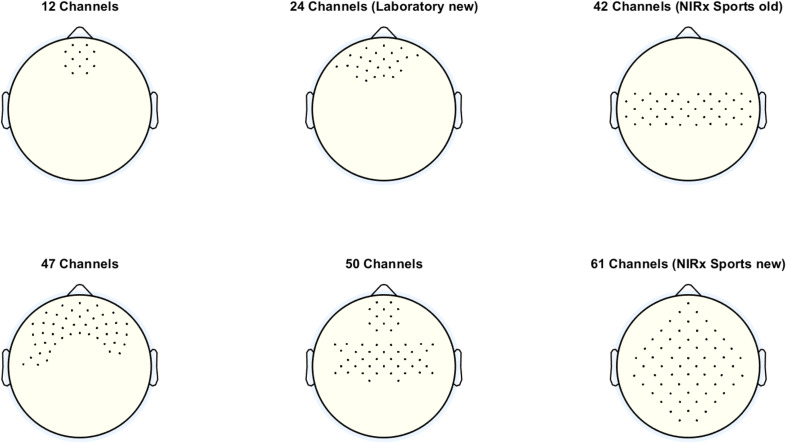
Different probe sets, which can be selected with the corresponding number and positions of the channels.

One very important setting at this panel is the *Probe Set*. With this setting, the user can decide how many channels are displayed in the oxy- and deoxy-Hb figures (concentration change signals and topoplots). The number of channels selected should match the number of channels used for recording the fNIRS signals. The number of channels used for recording depends on the optode setup, more precisely on the number of sources and detectors used, as well as their spatial distribution. Figure 2 shows the different probe sets which can be selected with the number and the positions of the channels.

##### Signal processing

In this panel, the signal analysis method for removing the physiological artifacts must be chosen (CAR, ICA, or TF models). The selection can be to remove all artifacts (MTH-waves, respiratory patterns, and pulsation of the heart) or just one or two of them (depending on which physiological signals were recorded during the measurement). If the option *Uncorrected* is chosen, no physiological artifact correction will be applied. Furthermore, different filters can be applied by clicking on the corresponding check boxes: Removing baseline drift (high-pass filter), a notch filter (removing 50 Hz power line interference) and a low-pass filter with its cut-off frequency (in Hz, to remove technical artifacts or instead of a physiological artifact correction method).

##### Physiological artifacts

In this section, the search windows and the correction band for each physiological artifact (in the frequency spectrum) are defined. Typically, the MTH waves are in a frequency range from 0.07 to 0.13 Hz, the RF from 0.2 to 0.4 Hz and the HR lies between 1 and 1.5 Hz (Bauernfeind et al., 2014). It is very important, that the search windows and the correction bands are chosen correctly, otherwise the physiological signals cannot be found in the frequency spectrum and therefore cannot be corrected. It is recommended to use the standard values as mentioned above, and to look at the frequency spectrum after the analysis. If a physiological artifact was not removed correctly, the search windows and correction bands must be adapted.

##### Timing

Here, the trials can be divided (as chosen from the condition marker) into a pre-task, a task and a post-task period. The different periods are separated in the analysis results figures (oxy-Hb and deoxy-Hb signals) by black vertical lines. These settings only apply for the figures of the ox-Hb and deoxy-Hb signals; the averaging and of the trials is done according to the condition markers.

##### Artifacts

If there the data is noisy of artifact afflicted (artifacts of technical origin), single trials or channels can be manually excluded from the measurement data for the current analysis. If the box for excluding a trial is checked, an input dialog box opens where the trial number must be inserted. The excluded channels must be selected at the panel *Channel* after clicking on the corresponding checkbox. Selecting the option *Consider Optode Failure* opens an input dialog box, where the channel number of the faulty optode has to be typed in, as well as the channels with which the faulty optode will be interpolated.

##### Channels

At this panel, figures of the oxy-Hb and deoxy-Hb signals for individual channels can be created by simply clicking on the corresponding check box. There are also checkboxes to select all channels at once (*All)* and to delete the selection of the channels again (*None)*. If the option *Exclude Channels* is selected, the channels to exclude can be chosen by the same way. With the radio buttons at *Channel Options*, it can be switched between settings of the channels to display and the channels to exclude.

##### Figure options

To visualize the analysis results, different types of figures can be generated. It is possible to create a figure with the physiological signals (biosignals figures) BP, RF, HR, and ECG, generate frequency spectrum figures (before and after artifact correction) and concentration change figures of the oxy-Hb and deoxy-Hb signals (optional including the standard deviation of the oxy-Hb and deoxy-Hb signals). Furthermore, a 2-D topographical map (topoplot) can be created, representing the changes of oxy-Hb and deoxy-Hb over the scalp. For that plot, the number of figures can be chosen, in order to show the topological concentration changes in specific time-points. The limits of the concentration range (in mM × mm) can also be set manually.

#### Starting the Analysis

If the measurement data is loaded, the analysis path selected and the analysis settings adjusted correctly, the text *Ready to start Analysis* will be displayed at the panel *Analysis Status* (Figure 1C). The analysis can then be started by clicking on the push button *Start Analysis* (Figure 1D). During the analysis, information about the individual analysis and signal processing steps will be provided at the panel *Output Text* (Figure 1E).

#### Error Handling

**FIGURE 3:**
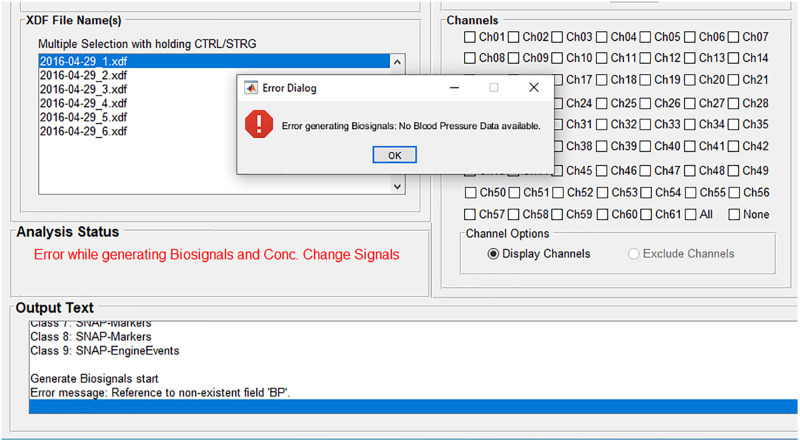
Error handling: The source of the error is displayed at the panel *Analysis Status;* more detailed information is displayed at the panel *Output Text* and at the popup window *Error Dialog*.

If an error occurs during an analysis, for example if a certain physiological signal necessary for the physiological artifact correction is not available, then the user gets informed about the error (Figure 3). The source of the error is displayed at the panel *Analysis Status* and the font color changes to red. More detailed information is displayed at the panel *Output Text* and at the popup window *Error Dialog*. In this example, the user is informed that there is no blood pressure data available, which would be displayed in the biosignals figure and used for the physiological artifact correction. In that way, the user knows how to avoid the error or how to adapt the analysis. Furthermore, the user is also informed if analysis settings are incomplete, incorrect (for example, a string instead of a value), or they do not match the dataset.

#### Grand Average Analysis

After all single subject analyses of a study are completed, it is possible to perform a grand average analysis of all the study subjects (group-level analysis). To apply this setting, the option *Start* at the menu bar entry *Grand Average* (Figure 1F) must be selected. After clicking on Start, the push button *Start Analysis* changes its name to *Start Grand Average*. Again, an analysis path for the new output files has to be defined. It is recommended to choose the same path as for the single subject analysis files.

If results of individual channels are preferred to be combined, it is possible to define regions of interest (ROIs). In order to do that, the number of ROIs must be defined and then the corresponding channels assigned to each ROI. This can be done by clicking on *Define ROIs* at the menu bar entry *Grand Average*.

## Results

### Description of the Output Files

During an analysis of fNIRS data with NICA, several output files and figures are created, which are saved automatically to the generated file path after the analysis. The folder structure is as follows: *Name of the study/Subject ID/Run number/Condition*

Depending on the selected figure options, figures with the physiological signals (BP, RF, HR, and ECG), frequency spectrum figures (all and/or single channels), concentration changes of oxy-Hb and deoxy-Hb (all and/or single channels) and a 2-D topographical map (topoplot), representing the changes of oxy-Hb and deoxy-Hb over the scalp, can be generated by the toolbox. The values of the concentration changes of oxy-Hb and deoxy-Hb, for each channel at each time point, are saved in a Microsoft Excel file, where they can be used for further statistical analysis. Furthermore, a MATLAB-file with all workspace variables is generated and the signal processing details of the analysis are saved in a text-file. All output files have the subject ID, the task name and the condition as prefix; the figure files have additionally the artifact correction method as suffix. Table 2 lists all output files which can be generated with NICA (without pre- and suffix).

**TABLE 2:** Generated output files with file format and a description of their content.

**File name**	**File format**	**Content**
Channels_Average	MATLAB-figure (.fig)	Concentration changes of oxy- and deoxy-Hb, averaged over trials, all channels
Conc_Chg_Avg_Ch#	MATLAB-figure (.fig)	Concentration changes of oxy- and deoxy-Hb, averaged over trials, single channel
Conc_Chg_Raw_Ch#	MATLAB-figure (.fig)	Concentration changes of oxy- and deoxy-Hb, continuous signal with condition markers
NIRx_Data	MATLAB-file (.mat)	All variables from the MATLAB workspace
Physio_Signals	MATLAB-figure (.fig)	Physiological signals (BP, RF, HR, ECG), continuous signal
Physio_Signals_Mean	MATLAB-figure (.fig)	Diastolic and systolic BP and HRV, averaged over trials
Signal_deoxy	Microsoft Excel (.xlsx)	Concentration change values of deoxy-Hb, for each channel at each time point
Signal_oxy	Microsoft Excel (.xlsx)	Concentration change values of oxy-Hb, for each channel at each time point
Spectra_Clean	MATLAB-figure (.fig)	Frequency spectrum of the fNIRS signal, averaged over trials, all channels, after artifact correction
Spectra_Compared	MATLAB-figure (.fig)	Frequency spectrum of the fNIRS signal, averaged over trials and channels, before and after artifact correction
Spectra_Raw	MATLAB-figure (.fig)	Frequency spectrum of the fNIRS signal, averaged over trials, all channels, before artifact correction
Text_Output	Text-file (.txt)	Information about the workflow and the signal processing steps
Topoplot	MATLAB-figure (.fig)	Concentration changes of oxy- and deoxy-Hb, averaged over trials, divided into time intervals, color maps

### Output Figures

In this section, the output figures generated with NICA are described on the basis of the example analysis of section “Performing an Analysis”.

**FIGURE 4:**
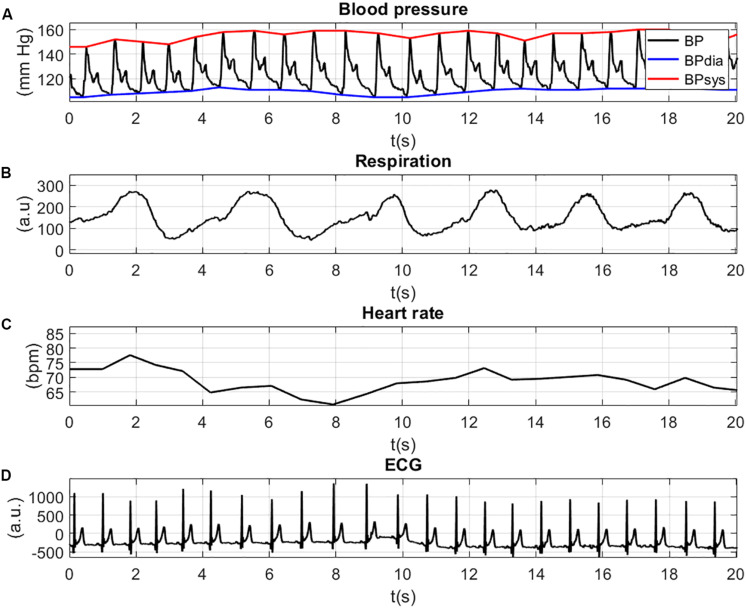
Physiological signals: BP waves with systolic (red line, BPsys) and diastolic (blue line, BPdia) values **(A)**; movement of the thorax due to respiration **(B)**; HR values **(C)**; and peaks of the ECG signal **(D)**.

The physiological signals are illustrated in Figure 4. For a better view of the details and the shape of the signals, the figure is zoomed in at a time-window of 20 s. The inspection of the figures can help to validate the values of the physiological signals. Figure 4A shows the BP waves with systolic (red line, BPsys) and diastolic (blue line, BPdia) values (in mm Hg). Both the diastolic BP values (>100 mm Hg) and the systolic BP values (>140 mm Hg) indicate high blood pressure (Hypertension). The movement of the thorax due to respiration is shown in Figure 4B and shows periodic extension and contraction of the respiration belt. The HR values (Figure 4C) lie between 60 and 80 bpm, which indicates a normal resting HR for adults. The patterns of the ECG signal with positive R-peaks and T-waves can be seen in Figure 4D.

**FIGURE 5:**
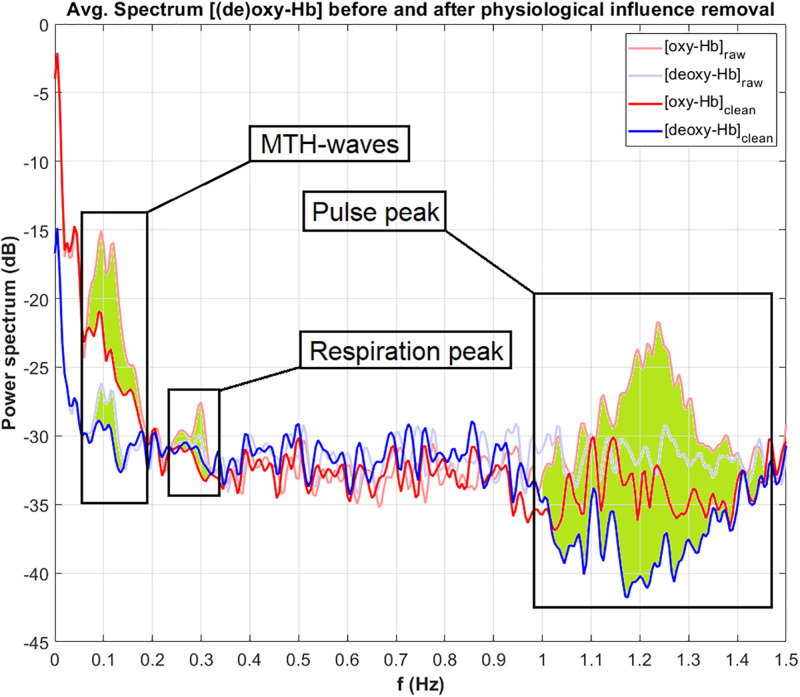
Power of the frequency spectrum of oxy-Hb (red line) and deoxy-Hb (blue line) signals, before (thin line, raw) and after (thick line, clean) artifact correction, averaged over all channels. The corrected frequency bands are highlighted in green.

Figure 5 displays the power (in dB) of the frequency spectrum of the oxy-Hb (red line) and deoxy-Hb (blue line) signals, before (raw, thick lines) and after (clean, thin lines) artifact correction, averaged over all channels. The corrected frequency bands are highlighted in green. The peak of the MTH-waves between 0.05 and 0.2 Hz was detected and removed, as well as the peaks of the RF at around 0.3 Hz and the influence of the pulsation of the heart between 1 and 1.5 Hz.

**FIGURE 6:**
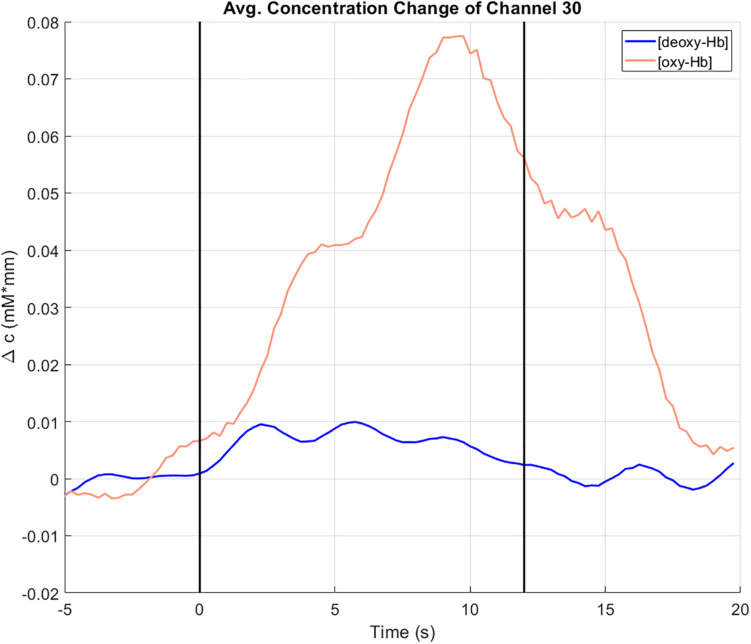
Concentration change of oxy-Hb (red) and deoxy-Hb (blue) at channel 30 (motor cortex, left hemisphere), averaged over all trials. The vertical lines at *t* = 0 and *t* = 12 indicate the start and the end of the right-hand ME task.

The concentration changes (in mM^∗^mm) of oxy-Hb (red line) and deoxy-Hb (blue line) at channel 30, averaged over all trials can be seen in Figure 6. Channel 30 is located on the left hemisphere at the primary motor cortex (Brodmann area 4). The vertical lines at *t* = 0 and *t* = 12 indicate the start and the end of the right-hand ME task. During the task, there is an increase of the concentration of oxy-Hb accompanied by slight increase of the concentration of deoxy-Hb. Toward the end of the task, both concentrations of oxy-Hb and deoxy-Hb start decreasing again. These concentration changes show a typical behavior that indicates cortical activation (Strangman et al., 2002; Obrig and Villringer, 2003).

**FIGURE 7:**
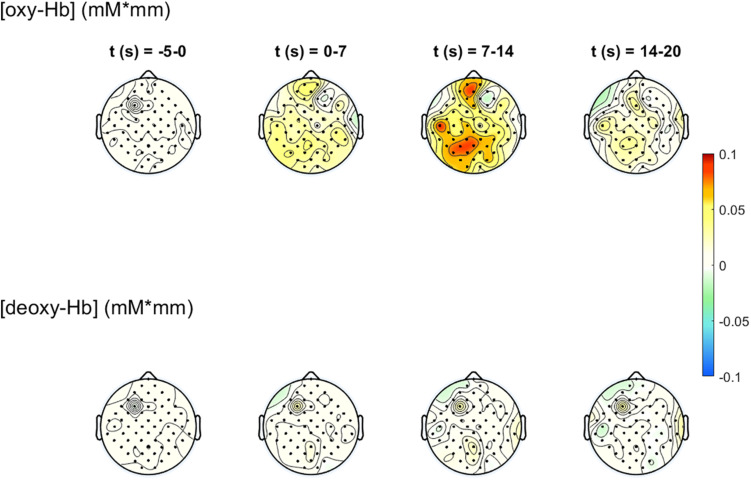
2-D topographical maps, representing the changes of oxy-Hb (top row) and deoxy-Hb (bottom row) over the scalp, divided into four time-ranges (*t*1 = −5 to 0 s, *t*2 = 0–7 s, *t*3 = 7–14 s, *t*4 = 14–20 s). The colors orange/red indicate an increase of the concentration, green/blue a decrease.

The 2-D topographical maps (topoplots), representing the changes of oxy-Hb (top row) and deoxy-Hb (bottom row) over the scalp, are shown in Figure 7. The concentration changes are divided into four time-windows (*t*1 = -5 to 0 s, *t*2 = 0–7 s, *t*3 = 7–14 s, *t*4 = 14–20 s). The colors orange/red indicate an increase of the concentration, green/blue a decrease. There is an increase of the concentration of oxy-Hb in the frontal and the parietal, motor related areas during the task, and only small changes in the concentration of deoxy-Hb. The highest increase in the concentration of oxy-Hb can be found at window *t*3, 7–14 s after task onset. These concentration changes corroborate the findings in Figure 6.

In addition to the figures shown in this section, the frequency spectra and the concentration changes of oxy-Hb and deoxy-Hb of each individual channel can be generated as well.

## Discussion

With NICA, a novel MATLAB toolbox for near-infrared spectroscopy calculations and analyses, it is possible to process and visualize fNIRS data in a straightforward and user-friendly way, as well as to apply physiological artifact correction methods. All output files and figures are saved automatically after an analysis in the generated analysis directory. Additionally, a proper error handling is implemented supporting an easy use of the toolbox. The surface of the GUI is divided into panels, which make it easy for the user to set and change different analysis settings that can also be saved and loaded for individual studies. Furthermore, NICA provides a comprehensive documentation of the analysis, which allows the user to follow and understand the individual signal processing steps.

For the correction of the physiological artifacts, three methods are implemented and described: CAR, ICA and TF models. According to Bauernfeind et al. (2014) using TF models with diastolic blood pressure as MTH-waves source is the most effective artifact correction method. However, it is still possible that in some cases the other methods can be more successful and lead to better results. In the example analysis in section “Performing an Analysis,” the best artifact correction method was TF models using the HR as MTH-waves source. It can be seen in Figure 5, that the physiological artifacts were successfully corrected. Nevertheless, it has to be mentioned that the artifact correction strongly depends on the signal quality of the physiological data. The correction of an artifact can only be as good as the recorded signal of this artifact.

The recorded data (fNIRS and physiological signals and paradigm markers) must be stored in an XDF-file, which is generated with the LSL recording software LabRecorder. It is possible to perform an analysis with fNIRS data exclusively (without additional physiological signals), but an artifact correction is only possible with the CAR method or with applying a low-pass filter then. Another important aspect is that the paradigm markers are set and recorded correctly. Without the markers it is not possible to divide the fNIRS signal into epochs and average it over trials or to distinguish between different conditions.

A limitation of NICA is the compatibility with different measurement systems. The software was developed for fNIRS data recorded with the NIRScout 1624 device. Although options for analyzing fNIRS data recorded with different measurement systems are available in NICA, they have not been tested or evaluated yet. Since the source code of the toolbox is freely available, it is possible to implement further or adapt existing functions.

## Data Availability Statement

The datasets generated for this study can be found in the authors GitHub repository https://github.com/praggam/NICA.

## Ethics Statement

Secondary data was used in this study from a preliminary study of Wriessnegger et al. (2017). This original study was approved by the local ethics committee (Medical University of Graz) and is in accordance with the ethical standards of the Declaration of Helsinki. After a detailed written and oral instruction, they gave informed written consent to participate in the study.

## Author Contributions

PR and SW developed the structure and the content of the manuscript. PR wrote the text of the manuscript and made the figures. SW supervised the toolbox. GB wrote parts of the functions used in the toolbox. SW and GB proofread and corrected the manuscript. All authors read and approved the final manuscript.

## Conflict of Interest

The authors declare that the research was conducted in the absence of any commercial or financial relationships that could be construed as a potential conflict of interest.

## Footnotes

1 https://github.com/praggam/NICA
